# An approach to $(\mu ,\nu ,\omega )$-single-valued neutrosophic submodules

**DOI:** 10.1038/s41598-022-18500-5

**Published:** 2023-01-14

**Authors:** Muhammad Shazib Hameed, Zaheer Ahmad, Shahbaz Ali, Muhammad Kamran, Alphonse-Roger Lula Babole

**Affiliations:** 1 Institute of Mathematics, Khwaja Fareed University of Engineering & Information Technology, Rahim Yar Khan, 64200 Punjab Pakistan; 2 Department of Mathematics, The Islamia University of Bahawalpur, Rahim Yar Khan Campus, 64200 Punjab Pakistan; 3 Department of Mathematics, Thal University, Bhakkar, 30000 Punjab Pakistan; 4 Department of Mathematics and Computer Science, University of Kinshasa, Kinshasa, Congo

**Keywords:** Engineering, Mathematics and computing

## Abstract

Dealing with erroneous, unexpected, susceptible, flawed, vulnerable, and intricate information is simplified with the use of a single-valued neutrosophic set (svns). This is because of the fact that these types of information are more sensitive to error. This is due to the fact that these particular kinds of information are more prone to error. The ideas of fuzzy sets and intuitionistic fuzzy sets have both undergone further development as a direct result of the development of this new theory. In svns, indeterminacy is quantified in a way that is both obvious and unambiguous, and truth membership, indeterminacy membership, and falsity membership are all completely independent of one another. In algebraic analysis, certain binary operations can be thought of as interacting with algebraic modules. These modules are intricate and ubiquitous structures. There are many different applications for modules to be used in. Modules find use in an extremely wide variety of different kinds of businesses and market segments. We investigate the idea of $(\mu ,\nu ,\omega )$-svns and relate it to $(\mu ,\nu ,\omega )$-single-valued neutrosophic module and $(\mu ,\nu ,\omega )$-single-valued neutrosophic submodule, respectively. The goals of this research are to comprehend the algebraic structures of a $(\mu ,\nu ,\omega )$-single-valued neutrosophic submodule of a classical module and enhance the legitimacy of this technique by discussing numerous essential aspects. Both of these goals will be accomplished through the course of this study. The strategies that we have developed in this manuscript are more generalizable than those that have been utilized in the past. These strategies include fuzzy sets, intuitionistic fuzzy sets, and neutrosophic sets.

## Introduction

We are expressing certain symbols in the Table 1, which will be used throughout this manuscript.

**Table 1:** Symbols and description of this manuscript.

Symbol	Description	Symbol	Description
svns	Single-valued neutrosophic set	svnss	Single-valued neutrosophic sets
${(\mu ,\nu ,\omega )}$-svns	${(\mu ,\nu ,\omega )}$-single-valued neutrosophic set	${(\mu ,\nu ,\omega )}$-svnss	${(\mu ,\nu ,\omega )}$-single-valued neutrosophic sets
${(\mu ,\nu ,\omega )}$-svnm	${(\mu ,\nu ,\omega )}$-single-valued neutrosophic module	${(\mu ,\nu ,\omega )}$-svnsm	${(\mu ,\nu ,\omega )}$-single-valued neutrosophic submodule
M	Classical module	R	Commutative ring with unity

The limitations of previously established fuzzy algebra structures are eradicated by the implementation of a recently proposed fuzzy algebraic structure. The use of regular mathematics is not always possible in many aspects of day-to-day life because of the prevalence of ambiguity and uncertainty in those settings. The application of a number of fuzzy algebraic structures, such as fuzzy subgroups, fuzzy rings, fuzzy sub-fields, and fuzzy sub-modules, can be very helpful in resolving issues of this nature. The ideas of the fuzzy set and the intuitionistic fuzzy set are expanded upon by use of svns, which is a powerful and comprehensive formal framework.

In the year 1980, Smarandache established the subfield of philosophy that is today known as neutrosophy. It is the cornerstone upon which such fields as neutrosophic logic, probability, set theory, and statistical analysis are built. As a consequence of this, he came up with the theory of neutrosophic logic and set, which provides an approximation of every statement of neutrosophic logic with the benefits of truth in the subcategory T, indeterminacy value in a subcategory I, and falsehood in the subcategory F. In light of the fact that the fuzzy set theory can only be used to depict situations in which there is uncertainty, the neutrosophic theory is the only viable option for describing scenarios in which there is indeterminacy. In^1^, Smarandache provided an explanation of the neutrosophic idea, and in^2,3^, Wang provided additional information on single-valued neutrosophic sets.

Researchers have already done extensive research on fuzzy and intuitionistic fuzzy sets^4–7^, fuzzy logics^8–10^, paraconsistent sets^11,12^, fuzzy groups^13–16^, complex fuzzy sets^17–19^, fuzzy subrings and ideals^20–26^, single-valued neutrosophic graphs and lattices^27–29^, single-valued neutrosophic algebras^30,31^ and many more interesting fields.

The neutrosophic theory ultimately led to the development of the algebraic neutrosophic structural principle. Kandasamy and Smarandache described shifts in the paradigm of algebraic structure theory in their paper which may be found in^1,2^. The term “svns” is used to characterize them in addition to the terms “algebraic structures” and “topological structures”^32–34^. This concept was utilized by Çetkin, Aygün, and Çetkin in the context of neutrosophic subgroups^35^, neutrosophic subrings^36^, and neutrosophic submodules^37,38^ of a certain classical group, ring, and module. Several recent research on the process of group decision making with a variety of different characteristics are described in^39–41^.

In actuality, modules are one of the most fundamental and diverse algebraic structures studied in terms of a number of binary operations. In this study, we investigate the concept of $(\mu ,\nu ,\omega )$-single-valued neutrosophic submodules, as well as the suggested concepts basic properties and characterizations. We also demonstrate that svns may not be an svnsm of module M, but $(\mu ,\nu ,\omega )$-svns must be an svnsm of module M. The essay is organised as follows: in “Preliminaries” section, we explain several basic ideas for svns. The “[Sec Sec3]” section explains the concept of $(\mu ,\nu ,\omega )$-svnsm and some idealistic findings.

## Preliminaries

This section covers basic definitions related to svns. In this section, we also present fundamental properties and relationships between svnss.

### **Definition 2.1**

^1^ On the universe set *X* a svns *P* is defined as:

$$\begin{aligned} P= \{\langle {m},~T_{P}{(m)},~I_{P}{(m)},~F_{P}{(m)}\rangle ,~m\in X\}, \end{aligned}$$

where $$T,I,F:X\rightarrow [0,1],$$ and $$0\le T_{P}{(m)}+ I_{P}{(m)}+F_{P}{(m)}\le 3$$, $$\forall ~m \in X$$, $$T_{P}{(m)}, I_{P}{(m)}, F_{P}{(m)} \in [0, 1]$$.

$T_{P}$, $I_{P}$, $F_{P}$ represents the functions of truth, indeterminacy, and falsity-membership, respectively.

### **Definition 2.2**

^2^ Suppose *P* and *Q* be two svnss on *X* and *Y*. The cartesian product of *P* and *Q* is then a svns on $X \times Y$, denoted by $P ~\times ~ Q$ and it is defined as:

$$\begin{aligned} (P ~\times ~ Q)(m, n) = P{(m)} ~\times ~ Q(n), \end{aligned}$$

where

$$\begin{aligned} P{(m)} ~\times ~ Q(n) =(T_{P ~\times ~ Q}(m, n),~I_{P ~\times ~ Q}(m, n), ~F_{P ~\times ~ Q}(m, n)), \end{aligned}$$

That is,

$$\begin{aligned} T_{P ~\times ~ Q}(m, n)&= T_{P}{(m)} ~\wedge ~ T_{Q}(n),\\ I_{P ~\times ~ Q}(m, n)&= I_{P}{(m)}~\wedge ~ I_{Q}(n),~~and\\ F_{P~\times ~ Q}(m, n)&= F_{P}{(m)}~\vee ~ F_{Q}(n). \end{aligned}$$

### **Definition 2.3**

^35^ Let *P* be a svns on *X* and $\alpha \in [0, 1]$. The $\alpha$-level sets on *P* can be determined:

$$\begin{aligned} (T_{P})_{\alpha }&= \{m\in X~|~T_{P}{(m)} \ge \alpha \},\\ (I_{P})_{\alpha }&= \{m\in X~|~I_{P}{(m)} \ge \alpha \},~and\\ (F_{P})^{\alpha }&= \{m\in X~|~F_{P}{(m)} \le \alpha \}. \end{aligned}$$

### **Proposition 2.4**

^2^ Let the svnss on the common universe *X* be *P*, *Q* and *S*. Then the following conditions must hold.

1. $$P\cup Q = Q\cup P, ~P\cap Q = Q\cap P$$.
2. $$P\cup (Q\cup S) = (P\cup Q)\cup S, ~P\cap (Q\cap S) = (P\cap Q)\cap S$$.
3. $$P\cup (Q\cap S) = (P\cup Q)\cap (P\cup S), ~P\cap (Q\cup S) = (P\cap Q)\cup (P\cap S)$$.
4. $$P\backslash (Q\cap S) = (P\backslash Q)\cup (P\backslash S), ~P\backslash (Q \cup S) = (P\backslash Q)\cap (P\backslash S)$$.
5. $c(P\cup Q)=c(P)\cap c(Q),~c(P\cap Q)=c(P)\cup c(Q)$. Note: c(P) represent complement of P.

### **Definition 2.5**

^2^ Let *P* & *Q* be two single-valued neutrosophic sets (svnss) on *X*. Then

1. $P\subseteq Q$, if and only if $P{(m)} \le Q{(m)}$.
  That is,
  $$\begin{aligned} T_{P}{(m)} \le T_{Q}{(m)},~I_{P}{(m)} \le I_{Q}{(m)},~and~F_{P}{(m)} \ge F_{Q}{(m)}. \end{aligned}$$
  Also $P=Q$ if and only if $P \subseteq Q$ and $Q \subseteq P$.
2. $$P\cup Q = \{ \langle \max \{T_{P}{(m)}, T_{Q}{(m)}\}, \max \{I_{P}{(m)}, I_{Q}{(m)}\}, \min \{F_{P}{(m)}, F_{Q}{(m)}\}\rangle , \forall ~m\in X\}.$$
3. $$P\cap Q = \{ \langle \min \{T_{P}{(m)}, T_{Q}{(m)}\}, \min \{I_{P}{(m)}, I_{Q}{(m)}\}, \max \{F_{P}{(m)}, F_{Q}{(m)}\}\rangle , \forall ~m\in X\}.$$
4. $$(P\backslash Q)=\{ \langle \min \{T_{P}{(m)}, T_{Q}{(m)}\}, \min \{I_{P}{(m)}, I_{Q}{(m)}\}, \max \{F_{P}{(m)}, F_{Q}{(m)}\rangle ,\forall ~m\in X\}.$$
5. $$c(P) = \{ \langle F_{P}{(m)}, 1-I_{P}{(m)}, T_{P}{(m)}, \forall ~m\in X \rangle \}.$$ Here $$c(c(P)) = P$$.

### **Definition 2.6**

^35^ Let define a function $g : X_{1} \longrightarrow X_{2}$ and *P*,  *Q* be the svnss of $X_{1}$ and $X_{2}$, respectively. Then the image of a svns *P* is also a svns of $X_{2}$ and as described below:

$$\begin{aligned} g(P)(n)&= (T_{g(P)}(n), I_{g(P)}(n), F_{g(P)}(n)\\&= (g(T_{P})(n), g(I_{P})(n), g(F_{P})(n)),~~\forall ~n\in X_{2}. \end{aligned}$$

Where

$$\begin{aligned} \ g(T_{P})(n)= \left\{ \begin{array}{ll} \bigvee T_{P}{(m)}, &{}~ {\text { if}} ~ m\in g^{-1}(n),\\ 0, &{}~ {\text {otherwise.}} \end{array} \right. \\ \ g(I_{P})(n)= \left\{ \begin{array}{ll} \bigvee I_{P}{(m)}, &{}~ {\text { if}} ~ m\in g^{-1}(n),\\ 0, &{}~ {\text {otherwise.}} \end{array} \right. \\ \ g(F_{P})(n)= \left\{ \begin{array}{ll} \bigwedge F_{P}{(m)}, &{}~ {\text { if}} ~ m\in g^{-1}(n),\\ 1, &{}~ {\text {otherwise.}} \end{array} \right. \end{aligned}$$

The preimage of a svns *Q* is a svns of $X_{1}$ and defined as:

$$\begin{aligned} g^{-1}(Q){(m)}&= (T_{g^{-1}(Q)}{(m)}, I_{g^{-1}(Q)}{(m)}, F_{g^{-1}(Q)}{(m)}\\&= (T_{Q}(g{(m)}), I_{Q}(g{(m)}), F_{Q}(g{(m)}))\\&= B(g{(m)}),~~\forall ~m\in X_{1}. \end{aligned}$$

## ${(\mu ,\nu ,\omega )}$-single-valued neutrosophic submodules

We define and investigate the basic properties and characterizations of a $(\mu ,\nu ,\omega )$-svnm and $(\mu ,\nu ,\omega )$-svnsm of a given classical module over a ring in this section. We typically start with some introductory $(\mu ,\nu ,\omega )$-svns, the cartesian product of $(\mu ,\nu ,\omega )$-svnss, the $\alpha$-level set on $(\mu ,\nu ,\omega )$-svns, operations and properties of $(\mu ,\nu ,\omega )$-svns, and then study crucial results, propositions, theorems and several examples related to $(\mu ,\nu ,\omega )$-svnm and $(\mu ,\nu ,\omega )$-svnsm of a given classical module over a ring *R*. In addition, we presented various homomorphism theorems for validity of $(\mu ,\nu ,\omega )$-svnsm.

### **Definition 3.1**

If *P* be a single-valued neutrosophic subset of *X* then ${(\mu ,\nu ,\omega )}$-single-valued neutrosophic subset *P* of *X* is categorize as:

$$\begin{aligned} P^{(\mu ,\nu ,\omega )}=\Big \{ \langle m,T_{P}^{\mu }{(m)},I_{P}^{\nu }{(m)},F_{P}^{\omega }{(m)}\rangle ~|~ m\in X\Big \}, \end{aligned}$$

where

$$\begin{aligned} T_{P}^{\mu }{(m)}&= \vee \{T_{P}{(m)}, \mu \},\\ I_{P}^{\nu }{(m)}&= \vee \{I_{P}{(m)},\nu \},\\ F_{P}^{\omega }{(m)}&= \wedge \{F_{P}{(m)}, \omega \}, \end{aligned}$$

such that

$$\begin{aligned} 0\le T_{P}^{\mu }{(m)}+ I_{P}^{\nu }{(m)}+F_{P}^{\omega }{(m)}\le 3. \end{aligned}$$

Where $\mu ,\nu ,\omega \in [0, 1]$, also $T,I,F: X\rightarrow [0,1]$, such that $T_{P}^{\mu }$, $I_{P}^{\nu }$, $F_{P}^{\omega }$ represents the functions of truth, indeterminacy, and falsity-membership, respectively.

### **Definition 3.2**

Let *X* be a space of objects, with *m* denoting a generic entity belong to *X*. A ${(\mu ,\nu ,\omega )}$-svns *P* on *X* is symbolized by truth $T_{P}^{\mu }$, indeterminacy $T_{P}^{\mu }$ and falsity-membership function $F_{P}^{\omega }$, respectively. For every *m* in *X*, $T_{P}^{\mu }(m), I_{P}^{\nu }(m), F_{P}^{\omega }(m) \in [0, 1]$, a ${(\mu ,\nu ,\omega )}$-svns *P* write accordingly as:

$$\begin{aligned} P^{(\mu ,\nu ,\omega )} =\sum \limits _{i}^{n}\langle T^{\mu }{(m_{i})}, I^{\nu }{(m_{i})}, F^{\omega }{(m_{i})} \rangle /m_{i},~ m_{i}\in X. \end{aligned}$$

### **Definition 3.3**

Let *P* and *Q* be two ${(\mu ,\nu ,\omega )}$-svnss on *X* and *Y*, respectively. Then the Cartesian product of $P^{(\mu ,\nu ,\omega )}$ and $Q^{(\mu ,\nu ,\omega )}$ which is denoted by $P^{(\mu ,\nu ,\omega )} \times Q^{(\mu ,\nu ,\omega )}$ is a ${(\mu ,\nu ,\omega )}$-svns on $X \times Y$ and it is defined as:

$$\begin{aligned} (P^{(\mu ,\nu ,\omega )} \times Q^{(\mu ,\nu ,\omega )})(m, n) = P^{(\mu ,\nu ,\omega )}{(m)} \times Q^{(\mu ,\nu ,\omega )}(n). \end{aligned}$$

where

$$\begin{aligned} P^{(\mu ,\nu ,\omega )}{(m)} \times Q^{(\mu ,\nu ,\omega )}(n)=(T_{P\times Q}^{\mu }(m, n),~I_{P\times Q}^{\nu }(m, n), ~F_{P\times Q}^{\omega }(m, n)). \end{aligned}$$

That is,

$$\begin{aligned} T_{P\times Q}^{\mu }(m, n)&= T_{P}^{\mu }{(m)}\wedge T_{Q}^{\mu }(n),\\ I_{P\times Q}^{\nu }(m, n)&= I_{P}^{\nu }{(m)}\wedge I_{Q}^{\nu }(n),\\ F_{P\times Q}^{\omega }(m, n)&= F_{P}^{\omega }{(m)}\vee F_{Q}^{\omega }(n). \end{aligned}$$

### **Definition 3.4**

Let *P* be a ${(\mu ,\nu ,\omega )}$-svns on *X* and $\alpha \in [0, 1]$. The $\alpha$-level sets on *P* can be determined as:

$$\begin{aligned} (T_{P}^{\mu })_{\alpha }&= \{m\in X~|~T_{P}^{\mu }{(m)} \ge \alpha \},\\ (I_{P}^{\nu })_{\alpha }&= \{m\in X~|~I_{P}^{\nu }{(m)} \ge \alpha \},\\ (F_{P}^{\omega })^{\alpha }&= \{m\in X~|~F_{P}^{\omega }{(m)} \le \alpha \}. \end{aligned}$$

### **Definition 3.5**

Let *P* and *Q* be two ${(\mu ,\nu ,\omega )}$-svnss on *X*. Then

1. $$P^{(\mu ,\nu ,\omega )}\subseteq Q^{(\mu ,\nu ,\omega )} ~~\Leftrightarrow P^{(\mu ,\nu ,\omega )}{(m)} \le Q^{(\mu ,\nu ,\omega )}{(m)}$$.
  That is,
  $$\begin{aligned} T_{P}^{\mu }{(m)}\le & {} T_{Q}^{\mu }{(m)},\\ I_{P}^{\nu }{(m)}\le & {} I_{Q}^{\nu }{(m)},\\ F_{P}^{\omega }{(m)}\ge & {} F_{Q}^{\omega }{(m)}, \end{aligned}$$
  and
  $$\begin{aligned} P^{(\mu ,\nu ,\omega )}& = Q^{(\mu ,\nu ,\omega )} \Leftrightarrow P^{(\mu ,\nu ,\omega )} \subseteq Q^{(\mu ,\nu ,\omega )} ~and~ Q^{(\mu ,\nu ,\omega )} \subseteq P^{(\mu ,\nu ,\omega )}. \end{aligned}$$
2. The union of $P^{(\mu ,\nu ,\omega )}$ and $Q^{(\mu ,\nu ,\omega )}$ is denoted by
  $$\begin{aligned} S^{(\mu ,\nu ,\omega )} = P^{(\mu ,\nu ,\omega )}~\cup ~ Q^{(\mu ,\nu ,\omega )}, \end{aligned}$$
  and defined as
  $$\begin{aligned} S^{(\mu ,\nu ,\omega )}{(m)} = P^{(\mu ,\nu ,\omega )}{(m)}~\vee ~ Q^{(\mu ,\nu ,\omega )}{(m)}, \end{aligned}$$
  where
  $$\begin{aligned} P^{(\mu ,\nu ,\omega )}{(m)}\vee Q^{(\mu ,\nu ,\omega )}{(m)} = \{ \langle T_{P}^{\mu }{(m)}\vee T_{Q}^{\mu }{(m)}, I_{P}^{\nu }{(m)}\vee I_{Q}^{\nu }{(m)}, F_{P}^{\omega }{(m)}\wedge F_{Q}^{\omega }{(m)} \rangle ,~ \forall ~ m\in X\}. \end{aligned}$$
  That is,
  $$\begin{aligned} T_{S}^{\mu }{(m)}&= \max \{T_{P}^{\mu }{(m)}, ~T_{Q}^{\mu }{(m)}\},\\ I_{S}^{\nu }{(m)}&= \max \{I_{P}^{\nu }{(m)}, ~I_{Q}^{\nu }{(m)}\},\\ F_{S}^{\omega }{(m)}&= \min \{F_{P}^{\omega }{(m)}, ~F_{Q}^{\omega }{(m)}\}. \end{aligned}$$
3. The intersection of $P^{(\mu ,\nu ,\omega )}$ and $Q^{(\mu ,\nu ,\omega )}$ is denoted by
  $$\begin{aligned} S^{(\mu ,\nu ,\omega )} = P^{(\mu ,\nu ,\omega )}\cap Q^{(\mu ,\nu ,\omega )}, \end{aligned}$$
  and defined as
  $$\begin{aligned} S^{(\mu ,\nu ,\omega )}{(m)} = P^{(\mu ,\nu ,\omega )}{(m)}\wedge Q^{(\mu ,\nu ,\omega )}{(m)}, \end{aligned}$$
  where
  $$\begin{aligned} P^{(\mu ,\nu ,\omega )}{(m)}\wedge Q^{(\mu ,\nu ,\omega )}{(m)} = \{ \langle T_{P}^{\mu }{(m)}\wedge T_{Q}^{\mu }{(m)}, ~I_{P}^{\nu }{(m)}\wedge I_{Q}^{\nu }{(m)},~F_{P}^{\omega }{(m)}\vee F_{Q}^{\omega }{(m)} \rangle , ~\forall ~ m\in X\}. \end{aligned}$$
  That is,
  $$\begin{aligned} T_{S}^{\mu }{(m)}&= \min \{T_{P}^{\mu }{(m)}, T_{Q}^{\mu }{(m)}\},\\ I_{S}^{\nu }{(m)}&= \min \{I_{P}^{\nu }{(m)}, I_{Q}^{\nu }{(m)}\},\\ F_{S}^{\omega }{(m)}&= \max \{F_{P}^{\omega }{(m)}, F_{Q}^{\omega }{(m)}\}. \end{aligned}$$
4. $$(P^{(\mu ,\nu ,\omega )}\backslash Q^{(\mu ,\nu ,\omega )})=\{ \langle \min \{T_{P}^{\mu }{(m)}, T_{Q}^{\mu }{(m)}\}, \min \{I_{P}^{\nu }{(m)}, I_{Q}^{\nu }{(m)}\}, \max \{F_{P}^{\omega }{(m)}, F_{Q}^{\omega }{(m)}\rangle ,\forall ~m\in X\}.$$
5. $$c(P^{(\mu ,\nu ,\omega )}) = \{ \langle (F_{P}^{\omega }{(m)}, 1-I_{P}^{\nu }{(m)}, T_{P}^{\mu }{(m)}), \rangle ,\forall ~m\in X \}.$$ Here, $$c(c(P^{(\mu ,\nu ,\omega )}) = P^{(\mu ,\nu ,\omega )}$$.

### **Proposition 3.6**

Let the ${(\mu ,\nu ,\omega )}$-svnss on the common universe *X* be *P*, *Q* and *S*. Then the following conditions must satisfy.

1. $$P^{(\mu ,\nu ,\omega )}\cup Q^{(\mu ,\nu ,\omega )} = Q^{(\mu ,\nu ,\omega )}\cup P^{(\mu ,\nu ,\omega )}, ~P^{(\mu ,\nu ,\omega )}\cap Q^{(\mu ,\nu ,\omega )} = Q^{(\mu ,\nu ,\omega )}\cap P^{(\mu ,\nu ,\omega )}$$.
2. $$P^{(\mu ,\nu ,\omega )}\cup (Q^{(\mu ,\nu ,\omega )}\cup S^{(\mu ,\nu ,\omega )}) = (P^{(\mu ,\nu ,\omega )}\cup Q^{(\mu ,\nu ,\omega )})\cup S^{(\mu ,\nu ,\omega )}$$, $$P^{(\mu ,\nu ,\omega )}\cap (Q^{(\mu ,\nu ,\omega )}\cap S^{(\mu ,\nu ,\omega )}) = (P^{(\mu ,\nu ,\omega )}\cap Q^{(\mu ,\nu ,\omega )})\cap S^{(\mu ,\nu ,\omega )}$$.
3. $$P^{(\mu ,\nu ,\omega )}\cup (Q^{(\mu ,\nu ,\omega )}\cap S^{(\mu ,\nu ,\omega )}) = (P^{(\mu ,\nu ,\omega )}\cup Q^{(\mu ,\nu ,\omega )})\cap (P^{(\mu ,\nu ,\omega )}\cup S^{(\mu ,\nu ,\omega )})$$, $$P^{(\mu ,\nu ,\omega )}\cap (Q^{(\mu ,\nu ,\omega )}\cup S^{(\mu ,\nu ,\omega )}) = (P^{(\mu ,\nu ,\omega )}\cap Q^{(\mu ,\nu ,\omega )})\cup (P^{(\mu ,\nu ,\omega )}\cap S^{(\mu ,\nu ,\omega )})$$.
4. $$P^{(\mu ,\nu ,\omega )}\backslash (Q^{(\mu ,\nu ,\omega )}\cap S^{(\mu ,\nu ,\omega )}) = (P^{(\mu ,\nu ,\omega )}\backslash Q^{(\mu ,\nu ,\omega )})\cup (P^{(\mu ,\nu ,\omega )}\backslash S^{(\mu ,\nu ,\omega )})$$, $$P^{(\mu ,\nu ,\omega )}\backslash (Q^{(\mu ,\nu ,\omega )} \cup S^{(\mu ,\nu ,\omega )}) = (P^{(\mu ,\nu ,\omega )}\backslash Q^{(\mu ,\nu ,\omega )})\cap (P^{(\mu ,\nu ,\omega )}\backslash S^{(\mu ,\nu ,\omega )})$$.
5. $$c(P^{(\mu ,\nu ,\omega )}\cup Q^{(\mu ,\nu ,\omega )})=c(P^{(\mu ,\nu ,\omega )})\cap c(Q^{(\mu ,\nu ,\omega )}),~c(P^{(\mu ,\nu ,\omega )}\cap Q^{(\mu ,\nu ,\omega )})=c(P^{(\mu ,\nu ,\omega )})\cup c(Q^{(\mu ,\nu ,\omega )})$$.

### **Definition 3.7**

Suppose a function $g : X_{1} \longrightarrow X_{2}$ and *P*,  *Q* be the two ${(\mu ,\nu ,\omega )}$-svnss of $X_{1}$ and $X_{2}$, respectively. Then the image of a ${(\mu ,\nu ,\omega )}$-svns $P^{(\mu ,\nu ,\omega )}$ is a ${(\mu ,\nu ,\omega )}$-svns of $X_{2}$ and it is defined as follows:

$$\begin{aligned} g(P^{(\mu ,\nu ,\omega )})(n)&= (T_{g(P)}^{\mu }(n), I_{g(P)}^{\nu }(n), F_{g(P)}^{\omega }(n))\\&= (g(T_{P}^{\mu })(n), g(I_{P}^{\nu })(n), g(F_{P}^{\omega })(n)), \forall ~n\in X_{2}. \end{aligned}$$

Where

$$\begin{aligned} \ g(T_{P}^{\mu })(n)= \left\{ \begin{array}{ll} \bigvee T_{P}^{\mu }{(m)}, &{}~ {\text { if}} ~ m\in g^{-1}(n),\\ 0, &{}~ {\text {otherwise.}} \end{array} \right. \\ \ g(I_{P}^{\nu })(n)= \left\{ \begin{array}{ll} \bigvee I_{P}^{\nu }{(m)}, &{}~ {\text { if}} ~ m\in g^{-1}(n),\\ 0, &{}~ {\text {otherwise.}} \end{array} \right. \\ \ g(F_{P}^{\omega })(n)= \left\{ \begin{array}{ll} \bigwedge F_{P}^{\omega }{(m)}, &{}~ {\text { if}} ~ m\in g^{-1}(n),\\ 1, &{}~ {\text {otherwise.}} \end{array} \right. \end{aligned}$$

The preimage of a ${(\mu ,\nu ,\omega )}$-svns *Q* is a ${(\mu ,\nu ,\omega )}$-svns of $X_{1}$ and defined as follows:

$$\begin{aligned} g^{-1}(Q^{(\mu ,\nu ,\omega )}){(m)}&= (T_{g^{-1}(Q)}^{\mu }{(m)}, I_{g^{-1}(Q)}^{\nu }{(m)}, F_{g^{-1}(Q)}^{\omega }{(m)})\\&= (T_{Q}^{\mu }(g{(m)}), I_{Q}^{\nu }(g{(m)}), F_{Q}^{\omega }(g{(m)}))\\&= Q^{(\mu ,\nu ,\omega )}(g{(m)}),~\forall ~m\in X_{1}. \end{aligned}$$

Note: We define and explore the notion of a $(\mu ,\nu ,\omega )$-svnsm of a given classical module M over a ring *R*. *R* is used throughout this article to represent a commutative ring with unity 1.

### **Definition 3.8**

Let *M* is a module over a ring *R*. A ${(\mu ,\nu ,\omega )}$-svns *P* on *M* is called a ${(\mu ,\nu ,\omega )}$-svnsm of *M* if the following conditions are satisfied:

- M1: $$P^{(\mu ,\nu ,\omega )}(0) = {\tilde{X}}$$. That is
  $$\begin{aligned} T_{P}^{\mu }(0) = 1,~I_{P}^{\nu }(0) = 1,~F_{P}^{\omega }(0) = 0. \end{aligned}$$
- M2:
  $$\begin{aligned} P^{(\mu ,\nu ,\omega )}(m + n) \ge P^{(\mu ,\nu ,\omega )}{(m)} \wedge P^{(\mu ,\nu ,\omega )}(n), \forall ~ m,n \in M. \end{aligned}$$
  That is,
  $$\begin{aligned} T_{P}^{\mu }(m + n)\ge & {} T_{P}^{\mu }{(m)}\wedge T_{P}^{\mu }(n),\\ I_{P}^{\nu }(m + n)\ge & {} I_{P}^{\nu }{(m)}\wedge I_{P}^{\nu }(n),\\ F_{P}^{\omega }(m + n)\le & {} F_{P}^{\omega }{(m)}\vee F_{P}^{\omega }(n). \end{aligned}$$
- M3:
  $$\begin{aligned} P^{(\mu ,\nu ,\omega )}(r m) \ge P^{(\mu ,\nu ,\omega )}{(m)}, \forall ~m\in M, r\in R. \end{aligned}$$
  That is,
  $$\begin{aligned} T_{P}^{\mu }(r m)\ge & {} T_{P}^{\mu }{(m)},\\ I_{P}^{\nu }(r m)\ge & {} I_{P}^{\nu }{(m)},\\ F_{P}^{\omega }(r m)\le & {} F_{P}^{\omega }{(m)}. \end{aligned}$$

$(\mu ,\nu ,\omega )$-svnsm(M) denotes the set of all $(\mu ,\nu ,\omega )$-single-valued neutrosophic submodules of *M*.

### **Example 3.9**

Take, for example, classical ring $R = Z_{4} = \{ {\bar{0}}, {\bar{1}}, {\bar{2}}, {\bar{3}}\}$. Since each ring is a module on itself, we consider $M = Z_{4}$ as a classical module. Define svns *P* as follows:

$$\begin{aligned} P = \{\langle 1, 1, 0 \rangle ~/{\bar{0}}+ \langle 0.3, 0.2, 0.8 \rangle ~/{\bar{1}}+ \langle 0.8, 0.5, 0.4 \rangle ~/{\bar{2}}+ \langle 0.2, 0.1, 0.7 \rangle ~/{\bar{3}}\}. \end{aligned}$$

It is clear that the svns *P* is a not a svnsm of the module *M*.

Let $\mu =0.6$, $\nu =0.3$ and $\omega =0.6$, So ${(\mu ,\nu ,\omega )}$-svns become

$$\begin{aligned} P = \{\langle 1, 1, 0 \rangle ~/{\bar{0}}+ \langle 0.6, 0.3, 0.6 \rangle ~/{\bar{1}}+ \langle 0.8, 0.5, 0.4 \rangle ~/{\bar{2}}+ \langle 0.6, 0.3, 0.6 \rangle ~/{\bar{3}}\}. \end{aligned}$$

It is clear that the ${(\mu ,\nu ,\omega )}$-svns *P* is a ${(\mu ,\nu ,\omega )}$-svnsm of the module $M = Z_{4}$.

### **Definition 3.10**

Let *P* be a ${(\mu ,\nu ,\omega )}$-svns on *M*, then $-P^{(\mu ,\nu ,\omega )}$ is a ${(\mu ,\nu ,\omega )}$-svns on *M*, defined as follows:

$$\begin{aligned} T_{-P}^{\mu }{(m)}&= T_{P}^{\mu }(-m),\\ I_{-P}^{\nu }{(m)}&= I_{P}^{\nu }(-m),\\ F_{-P}^{\omega }{(m)}&= F_{P}^{\omega }(-m),~~\forall ~m\in M. \end{aligned}$$

### Proposition 3.11

If *P* is a ${(\mu ,\nu ,\omega )}$-svnsm of an *R*-module *M*, then $(-1)P^{(\mu ,\nu ,\omega )}=-P^{(\mu ,\nu ,\omega )}$.

### *Proof*

Let $m\in M$ be arbitrary element

$$\begin{aligned} T_{(-1)P}^{\mu }{(m)}&= \bigvee \limits _{m=(-1)n}^{}T_{P}^{\mu }(n)\\&= \bigvee \limits _{n=-m}^{}T_{P}^{\mu }{(m)}= T_{P}^{\mu }{(-m)}\\&= T_{-P}^{\mu }{(m)}. \\ I_{(-1)P}^{\nu }{(m)}&= \bigvee \limits _{m=(-1)n}^{}I_{P}^{\nu }(n)\\&= \bigvee \limits _{n=-m}^{}I_{P}^{\nu }{(m)}=I_{P}^{\nu }{(-m)}\\&= I_{-P}^{\nu }{(m)} \\ F_{(-1)P}^{\omega }{(m)}&= \bigwedge \limits _{m=(-1)n}^{}F_{P}^{\omega }(n)\\&= \bigwedge \limits _{n=-m}^{}F_{P}^{\omega }{(m)}=F_{P}^{\omega }{(-m)}\\&= F_{-P}^{\omega }{(m)}. \end{aligned}$$

This shows that $T_{(-1)P}^{\mu }{(m)}=T_{-P}^{\mu }{(m)}$, $I_{(-1)P}^{\nu }{(m)} = I_{-P}^{\mu }{(m)}$ and $F_{(-1)P}^{\omega }{(m)} = F_{-P}^{\omega }{(m)}$.

Thus, this holds true for each $m\in M$,

$$\begin{aligned} (-1)P^{(\mu ,\nu ,\omega )} = (T_{(-1)P}^{\mu }, I_{(-1)P}^{\nu }, F_{(-1)P}^{\omega })= (T_{-P}^{\mu }, I_{-P}^{\nu }, F_{-P}^{\omega }) = -P^{(\mu ,\nu ,\omega )}. \end{aligned}$$

$$\square$$

### **Definition 3.12**

Let *P* be a ${(\mu ,\nu ,\omega )}$-svns on an *R*-module *M* with $r \in R$. Set *rP* as a neutrosophic set to *M*, define as:

$$\begin{aligned} T_{rP}^{\mu }{(m)}&= \vee \{T_{P}^{\mu }(n)~|~ n\in M,~m=r n\},\\ I_{rP}^{\nu }{(m)}&= \vee \{I_{P}^{\nu }(n)~|~ n\in M,~m=r n\},\\ F_{rP}^{\omega }{(m)}&= \wedge \{F_{P}^{\omega }(n)~|~ n\in M,~m=r n\}. \end{aligned}$$

### **Definition 3.13**

Let *P*,  *Q* be ${(\mu ,\nu ,\omega )}$-svnss on *M*. Then their sum

$P^{(\mu ,\nu ,\omega )} + Q^{(\mu ,\nu ,\omega )}$ is a ${(\mu ,\nu ,\omega )}$-svns on *M*, defined as follows:

$$\begin{aligned} T_{P+Q}^{\mu }{(m)}&= \vee \{ T_{P}^{\mu }(n) \wedge T_{Q}^{\mu }( o) ~|~ m = n + o, ~n, o \in M \},\\ I_{P+Q}^{\nu }{(m)}&= \vee \{ I_{P}^{\nu }(n) \wedge I_{Q}^{\nu }( o) ~|~ m = n + o, ~n, o \in M \},\\ F_{P+Q}^{\omega }{(m)}&= \wedge \{ F_{P}^{\omega }(n) \vee F_{Q}^{\omega }( o) ~|~ m = n + o, ~n, o \in M \}. \end{aligned}$$

### Proposition 3.14

If *P* and *Q* are ${(\mu ,\nu ,\omega )}$-svnss on *M* with $P^{(\mu ,\nu ,\omega )} \subseteq Q^{(\mu ,\nu ,\omega )}$, then $rP^{(\mu ,\nu ,\omega )}\subseteq rQ^{(\mu ,\nu ,\omega )}$, for each $r\in R$.

### *Proof*

By definition, it is obvious. $\square$

### Proposition 3.15

If *P* is ${(\mu ,\nu ,\omega )}$-svns on *M*, then $T^{\mu }_{rP}(r m) \ge T_{P}^{\mu }{(m)}, I^{\nu }_{rP}(r m) \ge I_{P}^{\nu }{(m)}$ and $F^{\omega }_{rP}(r m) \le F_{P}^{\omega }{(m)}$.

### *Proof*

By definition, it is obvious. $\square$

### Proposition 3.16

If *P* is a ${(\mu ,\nu ,\omega )}$-svns on *M*, then $r(sP^{(\mu ,\nu ,\omega )}) = (rs)P^{(\mu ,\nu ,\omega )}$, $\forall$ $r,~s\in R$.

### *Proof*

Consider $r, s\in R$ be arbitrary whereas $m\in M$.

$$\begin{aligned} T^{\mu }_{r(sP)}{(m)}&= \bigvee \limits _{m=r n}^{}T_{sP}^{\mu }(n)\\&= \bigvee \limits _{m=r n}^{}\bigvee \limits _{n=s t}^{}T_{P}^{\mu }( t)=\bigvee \limits _{m=r(s t)}^{}T_{P}^{\mu }( t)\\&= T^{\mu }_{(rs)P}{(m)}. \\ I^{\nu }_{r(sP)}{(m)}&= \bigvee \limits _{m=r n}^{}I_{sP}^{\nu }(n)\\&= \bigvee \limits _{m=r n}^{}\bigvee \limits _{n=s t}^{}I_{P}^{\nu }( t)=\bigvee \limits _{m=r(s t)}^{}I_{P}^{\nu }( t)\\&= I^{\nu }_{(rs)P}{(m)}. \\ F^{\omega }_{r(sP)}{(m)}&= \bigwedge \limits _{m=r n}^{}F_{sP}^{\omega }(n)\\&= \bigwedge \limits _{m=r n}^{}\bigwedge \limits _{n=s t}^{}F_{P}^{\omega }( t)=\bigwedge \limits _{m=r(s t)}^{}F_{P}^{\omega }( t)\\&= F^{\omega }_{(rs)P}{(m)}. \end{aligned}$$

So we have the following equalities

$$\begin{aligned} T^{\mu }_{r(sP)}{(m)}&= T^{\mu }_{(rs)P}{(m)},\\ I^{\nu }_{r(sP)}{(m)}&= I^{\nu }_{(rs)P}{(m)},\\ F^{\omega }_{r(sP)}{(m)}&= F^{\omega }_{(rs)P}{(m)}. \end{aligned}$$

Therefore,

$$\begin{aligned} r(sP^{(\mu ,\nu ,\omega )}) = (T^{\mu }_{r(sP)}, I^{\nu }_{r(sP)}, F^{\omega }_{r(sP)}), \end{aligned}$$

$$\Rightarrow r(sP^{(\mu ,\nu ,\omega )})= (T^{\mu }_{(rs)P}, I^{\nu }_{(rs)P}, F^{\omega }_{(rs)P}) = (rs)P^{(\mu ,\nu ,\omega )}.$$ $$\square$$

### Proposition 3.17

If *P* and *Q* are ${(\mu ,\nu ,\omega )}$-svnss on *M*, then

1. $T_{Q}^{\mu }(rm) \ge T_{P}^{\mu }{(m)}$, for each $m\in M$, if and only if $T^{\mu }_{rP} \le T_{Q}^{\mu }$.
2. $I_{Q}^{\nu }(rm) \ge I_{P}^{\nu }{(m)}$, for each $m\in M$, if and only if $I^{\nu }_{rP} \le I_{Q}^{\nu }$.
3. $F_{Q}^{\omega }(rm) \le F_{P}^{\omega }{(m)}$, for each $m\in M$, if and only if $F^{\omega }_{rP} \ge F_{Q}^{\omega }$.

### *Proof*

(1) Suppose $T_{Q}^{\mu }(r m) \ge T_{P}^{\mu }{(m)}$, for each $m\in M$, then

$$\begin{aligned} T^{\mu }_{rP}{(m)} = \bigvee \limits _{m=r n,n\in M}^{}T_{P}^{\mu }(n). \end{aligned}$$

So,

$$\begin{aligned} T^{\mu }_{rP} \le T_{Q}^{\mu }. \end{aligned}$$

Conversely, suppose $T^{\mu }_{rP} \le T_{Q}^{\mu }$. Then $T^{\mu }_{rP}{(m)} = T_{Q}^{\mu }{(m)}$, for each $m\in M$.

Hence,

$$\begin{aligned} T_{Q}^{\mu }(r m) \ge T^{\mu }_{rP}(r m) \ge T_{P}^{\mu }{(m)},~~\forall ~ m\in M ~~\text {(from Proposition~3.15)}. \end{aligned}$$

(2) Suppose $I_{Q}^{\nu }(r m) \ge I_{P}^{\nu }{(m)}$, for each $m\in M$, then

$$\begin{aligned} I^{\nu }_{rP}{(m)} = \bigvee \limits _{m=r n,n\in M}^{}I_{P}^{\nu }(n). \end{aligned}$$

So,

$$\begin{aligned} I^{\nu }_{rP} \le I_{Q}^{\nu }. \end{aligned}$$

Conversely, suppose $I^{\nu }_{rP} \le I_{Q}^{\nu }$. Then $I^{\nu }_{rP}{(m)} = I_{Q}^{\nu }{(m)}$, for each $m\in M.$

Hence,

$$\begin{aligned} I_{Q}^{\nu }(r m) \ge I^{\nu }_{rP}(r m) \ge I_{P}^{\nu }{(m)},~~\forall ~ m\in M~~ \text {(from Proposition~3.15)}. \end{aligned}$$

(3) Suppose $F_{Q}^{\omega }(r m) \le I_{P}^{\omega }{(m)}$, for each $m\in M$, then

$$\begin{aligned} F^{\omega }_{rP}{(m)} = \bigwedge \limits _{m=r n,n\in M}^{}F_{P}^{\omega }(n). \end{aligned}$$

So,

$$\begin{aligned} F^{\omega }_{rP} \ge F_{Q}^{\omega }. \end{aligned}$$

Conversely, suppose $F^{\omega }_{rP} \ge F_{Q}^{\omega }$. Then $F^{\omega }_{rP}{(m)} = F_{Q}^{\omega }{(m)}$, for each $m\in M.$

Hence,

$$\begin{aligned} F_{Q}^{\omega }(r m) \le F^{\omega }_{rP}(r m) \le F_{P}^{\omega }{(m)}, ~\forall ~ m\in M~~ \text {(using Proposition~3.15)}. \end{aligned}$$

$$\square$$

### Proposition 3.18

If *P* and *Q* are ${(\mu ,\nu ,\omega )}$-svnss on *M*, then $r(P^{(\mu ,\nu ,\omega )} + Q^{(\mu ,\nu ,\omega )}) = rP^{(\mu ,\nu ,\omega )} + rQ^{(\mu ,\nu ,\omega )},~\forall ~ r\in R.$

### *Proof*

Let *P* and *Q* are ${(\mu ,\nu ,\omega )}$-svnss on *M*, $m\in M$ and $r\in R$.

$$\begin{aligned} T^{\mu }_{r(P+Q)}{(m)}&= \bigvee \limits _{m=r n}^{}T^{\mu }_{(P+Q)}(n)\\&= \bigvee \limits _{m=r n}^{}\bigvee \limits _{n= t_{1}+ t_{2}}^{}(T_{P}^{\mu }( t_{1}) \wedge T_{Q}^{\mu }( t_{2}))\\&= \bigvee \limits _{m=r t_{1}+r t_{2}}^{}(T_{P}^{\mu }( t_{1}) \wedge T_{Q}^{\mu }( t_{2}))\\&= \bigvee \limits _{m=m_{1}+m_{2}}^{}(\bigvee \limits _{m_{1}=r t_{1}}^{}(T_{P}^{\mu }( t_{1}) \wedge \bigvee \limits _{m_{2}=r t_{2}}^{}T_{Q}^{\mu }( t_{2})))\\&= \bigvee \limits _{m=m_{1}+m_{2}}^{}(T^{\mu }_{rP}(m_{1}) \wedge T^{\mu }_{rQ}(m_{2}))\\&= T^{\mu }_{rP+rQ}{(m)}. \\ I^{\nu }_{r(P+Q)}{(m)}&= \bigvee \limits _{m=r n}^{}I^{\nu }_{(P+Q)}(n)\\&= \bigvee \limits _{m=r n}^{}\bigvee \limits _{n= t_{1}+ t_{2}}^{}(I_{P}^{\nu }( t_{1}) \wedge I_{Q}^{\nu }( t_{2}))\\&= \bigvee \limits _{m=r t_{1}+r t_{2}}^{}(I_{P}^{\nu }( t_{1}) \wedge I_{Q}^{\nu }( t_{2}))\\&= \bigvee \limits _{m=m_{1}+m_{2}}^{}(\bigvee \limits _{m_{1}=r t_{1}}^{}(I_{P}^{\nu }( t_{1}) \wedge \bigvee \limits _{m_{2}=r t_{2}}^{}I_{Q}^{\nu }( t_{2})))\\&= \bigvee \limits _{m=m_{1}+m_{2}}^{}(I^{\nu }_{rP}(m_{1}) \wedge I^{\nu }_{rQ}(m_{2}))\\&= I^{\nu }_{rP+rQ}{(m)}. \\ F^{\omega }_{r(P+Q)}{(m)}&= \bigwedge \limits _{m=r n}^{}F^{\omega }_{(P+Q)}(n)\\&= \bigwedge \limits _{m=r n}^{}\bigwedge \limits _{n= t_{1}+ t_{2}}^{}(F_{P}^{\omega }( t_{1}) \vee F_{Q}^{\omega }( t_{2}))\\&= \bigwedge \limits _{m=r t_{1}+r t_{2}}^{}(F_{P}^{\omega }( t_{1}) \vee F_{Q}^{\omega }( t_{2}))\\&= \bigwedge \limits _{m=m_{1}+m_{2}}^{}(\bigwedge \limits _{m_{1}=r t_{1}}^{}(F_{P}^{\omega }( t_{1}) \vee \bigwedge \limits _{m_{2}=r t_{2}}^{}F_{Q}^{\omega }( t_{2})))\\&= \bigwedge \limits _{m=m_{1}+m_{2}}^{}(F^{\omega }_{rP}(m_{1}) \vee F^{\omega }_{rQ}(m_{2}))\\&= F^{\omega }_{rP+rQ}{(m)}. \end{aligned}$$

So we have the equalities

$$\begin{aligned} T^{\mu }_{r(P+Q)}{(m)}&= T^{\mu }_{rP+rQ}{(m)},\\ I^{\nu }_{r(P+Q)}{(m)}&= I^{\nu }_{rP+rQ}{(m)},\\ F^{\omega }_{r(P+Q)}{(m)}&= F^{\omega }_{rP+rQ}{(m)}.\\ \end{aligned}$$

Hence,

$$\begin{aligned} r(P^{(\mu ,\nu ,\omega )} + Q^{(\mu ,\nu ,\omega )})&= (T^{\mu }_{r(P+Q)}, I^{\nu }_{r(P+Q)}, F^{\omega }_{r(P+Q)}) \\&= (T^{\mu }_{rP+rQ}, I^{\nu }_{rP+rQ}, F^{\omega }_{rP+rQ})\\&= rP^{(\mu ,\nu ,\omega )} + rQ^{(\mu ,\nu ,\omega )}. \end{aligned}$$

$$\square$$

### Proposition 3.19

If *P* and *Q* are ${(\mu ,\nu ,\omega )}$-svnss on *M*, then

1. $$T^{\mu }_{rP+sQ}(rm + sn) \ge T_{P}^{\mu }{(m)} \wedge T_{Q}^{\mu }(n)$$,
2. $$I^{\nu }_{rP+sQ}(rm + sn) \ge I_{P}^{\nu }{(m)} \wedge I_{Q}^{\nu }(n)$$,
3. $$F^{\omega }_{rP+sQ}(rm + sn) \le F_{P}^{\omega }{(m)} \vee F_{Q}^{\omega }(n)$$, for each $$m, n\in M$$, $$r, s \in R$$.

### *Proof*

It is easy to prove with the help of Definitions 3.13, 3.12 and Proposition 3.15. $\square$

### Example 3.20

Take an example for above Proposition 3.19, classical ring $R = Z_{2} = \{ {\bar{0}}, {\bar{1}}\}$. Since each ring is a module on itself, we consider $M = Z_{2}$ as a classical module. Define svnss *P* and *Q* as follows:

$$\begin{aligned} P = \{\langle 1, 1, 0 \rangle ~/{\bar{0}}+ \langle 0.6, 0.3, 0.6 \rangle ~/{\bar{1}} ~and~ Q=\{ \langle 1, 1, 0 \rangle ~/{\bar{0}}+ \langle 0.8, 0.1, 0.4 \rangle ~/{\bar{1}}\}. \end{aligned}$$

Let $\mu =0.6$, $\nu =0.3$ and $\omega =0.6$, So ${(\mu ,\nu ,\omega )}$-svnss *P* and *Q* becomes

$$\begin{aligned} P = \{\langle 1, 1, 0 \rangle ~/{\bar{0}}+ \langle 0.6, 0.3, 0.6 \rangle ~/{\bar{1}} ~and~ Q=\{ \langle 1, 1, 0 \rangle ~/{\bar{0}}+ \langle 0.8, 0.3, 0.4 \rangle ~/{\bar{1}}\}. \end{aligned}$$

We can examine that for truth-membership

$$T_{P}^{\mu }{(0)}=1$$, $$T_{P}^{\mu }{(1)}=0.6$$, $$T_{Q}^{\mu }{(0)}=1$$, $$T_{Q}^{\mu }{(1)}=0.8$$ and

$$T_{P}^{\mu }{(0)} \wedge T_{Q}^{\mu }{(0)}=1$$, $$T_{P}^{\mu }{(0)} \wedge T_{Q}^{\mu }{(1)}=0.8$$, $$T_{P}^{\mu }{(1)} \wedge T_{Q}^{\mu }{(0)}=0.6$$, and $$T_{P}^{\mu }{(1)} \wedge T_{Q}^{\mu }{(1)}=0.6$$.

Also we can see that

$$T_{rP}^{\mu }{(0)}=1$$, $$T_{rP}^{\mu }{(1)}=0.6$$, $$T_{sQ}^{\mu }{(0)}=1$$, $$T_{sQ}^{\mu }{(1)}=0.8$$ and $$T_{rP+sQ}^{\mu }{(0)}=1$$, $$T_{rP+sQ}^{\mu }{(1)}=0.8$$.

- Case 1: Let $m=0,~n=0$ and $r, s \in R=Z_{2}$, clearly $T^{\mu }_{rP+sQ}(r0 + s0)=1 \ge T_{P}^{\mu }{(0)} \wedge T_{Q}^{\mu }(0)=1$.
- Case 2: Let $m=0,~n=1$ and $r, s \in R=Z_{2}$, clearly $T^{\mu }_{rP+sQ}(r0 + s1)=1 ~or~ 0.8 \ge T_{P}^{\mu }{(0)} \wedge T_{Q}^{\mu }(1)=0.8$.
- Case 3: Let $m=1,~n=0$ and $r, s \in R=Z_{2}$, clearly $T^{\mu }_{rP+sQ}(r1 + s0)=1 ~or~ 0.8 \ge T_{P}^{\mu }{(1)} \wedge T_{Q}^{\mu }(0)=0.6$.
- Case 4: Let $m=1,~n=1$ and $r, s \in R=Z_{2}$, clearly $T^{\mu }_{rP+sQ}(r1 + s1)=1 ~or~ 0.8 \ge T_{P}^{\mu }{(1)} \wedge T_{Q}^{\mu }(0)=0.6$.

$\Rightarrow$ ${(\mu ,\nu ,\omega )}$-svnss *P* and *Q* satisfy the condition

(1) $$T^{\mu }_{rP+sQ}(rm + sn) \ge T_{P}^{\mu }{(m)} \wedge T_{Q}^{\mu }(n)$$,

Similarly we can show that for indeterminacy membership

(2) $$I^{\nu }_{rP+sQ}(rm + sn) \ge I_{P}^{\nu }{(m)} \wedge I_{Q}^{\nu }(n)$$,

Now, we prove for the falsity membership

$$F_{P}^{\mu }{(0)}=0$$, $$F_{P}^{\mu }{(1)}=0.6$$, $$F_{Q}^{\mu }{(0)}=0$$, $$F_{Q}^{\mu }{(1)}=0.4$$ and

$$F_{P}^{\mu }{(0)} \vee F_{Q}^{\mu }{(0)}=0$$, $$F_{P}^{\mu }{(0)} \vee F_{Q}^{\mu }{(1)}=0.4$$, $$F_{P}^{\mu }{(1)} \vee F_{Q}^{\mu }{(0)}=0.6$$, and $$F_{P}^{\mu }{(1)} \vee F_{Q}^{\mu }{(1)}=0.6$$.

Also we can see that

$$F_{rP}^{\mu }{(0)}=0$$, $$F_{rP}^{\mu }{(1)}=0.6$$, $$F_{sQ}^{\mu }{(0)}=0$$, $$F_{sQ}^{\mu }{(1)}=0.4$$ and $$F_{rP+sQ}^{\mu }{(0)}=0$$, $$F_{rP+sQ}^{\mu }{(1)}=0$$.

- Case 1: Let $m=0,~n=0$ and $r, s \in R=Z_{2}$, clearly $F^{\mu }_{rP+sQ}(r0 + s0)=0 \le F_{P}^{\mu }{(0)} \vee F_{Q}^{\mu }(0)=0$.
- Case 2: Let $m=0,~n=1$ and $r, s \in R=Z_{2}$, clearly $F^{\mu }_{rP+sQ}(r0 + s1)=0 \le F_{P}^{\mu }{(0)} \vee F_{Q}^{\mu }(1)=0.4$.
- Case 3: Let $m=1,~n=0$ and $r, s \in R=Z_{2}$, clearly $F^{\mu }_{rP+sQ}(r1 + s0)=0 \le F_{P}^{\mu }{(1)} \vee F_{Q}^{\mu }(0)=0.6$.
- Case 4: Let $m=1,~n=1$ and $r, s \in R=Z_{2}$, clearly $F^{\mu }_{rP+sQ}(r1 + s1)=0 \le F_{P}^{\mu }{(1)} \vee F_{Q}^{\mu }(0)=0.6$.

$\Rightarrow$ ${(\mu ,\nu ,\omega )}$-svnss *P* and *Q* satisfy the condition

(3) $$F^{\omega }_{rP+sQ}(rm + sn) \le F_{P}^{\omega }{(m)} \vee F_{Q}^{\omega }(n)$$, for each $$m, n\in M$$, $$r, s \in R$$.

### Proposition 3.21

If *P*, *Q*, *S* are ${(\mu ,\nu ,\omega )}$-svnss on *M*, Then, for each $r, s \in R$, the followings are satisfied;

1. $T^{\mu }_{S}(rm + sn) \ge T_{P}^{\mu }{(m)} \wedge T_{Q}^{\mu }(n)$, for all $m, n\in M$ if and only if $T^{\mu }_{rP+sQ} \le T^{\mu }_{S}$.
2. $I^{\nu }_{S}(rm + sn) \ge I_{P}^{\nu }{(m)} \wedge I_{Q}^{\nu }(n)$, for all $m, n\in M$ if and only if $I_{rP+sQ}^{\nu } \le I_{S}^{\nu }$.
3. $F_{S}^{\omega }(rm + sn) \le F_{P}^{\omega }{(m)} \vee F_{Q}^{\omega }(n)$, for all $m, n\in M$ if and only if $F_{rP+sQ}^{\omega } \ge F_{S}^{\omega }$.

### *Proof*

It is easy to prove with the help of Proposition 3.19. $\square$

### **Example 3.22**

Take an example for above Proposition 3.21, Let us take the classical ring $R = Z_{2} = \{ {\bar{0}}, {\bar{1}}\}$. Since each ring is a module on itself, we consider $M = Z_{2}$ as a classical module. Define svnss *P*, *Q* and *S* as follows:

$$\begin{aligned} P = \{\langle 1, 1, 0 \rangle ~/{\bar{0}}+ \langle 0.3, 0.2, 0.8 \rangle ~/{\bar{1}},~ Q=\{ \langle 1, 1, 0 \rangle ~/{\bar{0}}+ \langle 0.4, 0.5, 0.4 \rangle ~/{\bar{1}}\} ~and~ S=\{ \langle 1, 1, 0 \rangle ~/{\bar{0}}+ \langle 0.2, 0.1, 0.7 \rangle ~/{\bar{1}}\}. \end{aligned}$$

Let $\mu =0.6$, $\nu =0.3$ and $\omega =0.6$, So ${(\mu ,\nu ,\omega )}$-svnss *P*, *Q* and *S* becomes

$$\begin{aligned} P = \{\langle 1, 1, 0 \rangle ~/{\bar{0}}+ \langle 0.6, 0.3, 0.6 \rangle ~/{\bar{1}}, Q=\{ \langle 1, 1, 0 \rangle ~/{\bar{0}}+ \langle 0.6, 0.5, 0.4 \rangle ~/{\bar{1}}\} ~and~ S=\{ \langle 1, 1, 0 \rangle ~/{\bar{0}}+ \langle 0.6, 0.3, 0.6 \rangle ~/{\bar{1}}\}. \end{aligned}$$

We can see that for truth-membership

$$T_{P}^{\mu }{(0)}=1$$, $$T_{P}^{\mu }{(1)}=0.6$$, $$T_{Q}^{\mu }{(0)}=1$$, $$T_{Q}^{\mu }{(1)}=0.8$$, $$T_{S}^{\mu }{(0)}=1$$, $$T_{S}^{\mu }{(1)}=0.6$$ and

$$T_{P}^{\mu }{(0)} \wedge T_{Q}^{\mu }{(0)}=1$$, $$T_{P}^{\mu }{(0)} \wedge T_{Q}^{\mu }{(1)}=0.6$$, $$T_{P}^{\mu }{(1)} \wedge T_{Q}^{\mu }{(0)}=0.6$$, and $$T_{P}^{\mu }{(1)} \wedge T_{Q}^{\mu }{(1)}=0.6$$.

Also we can see that

$$T_{rP}^{\mu }{(0)}=1$$, $$T_{rP}^{\mu }{(1)}=0.6$$, $$T_{sQ}^{\mu }{(0)}=1$$, $$T_{sQ}^{\mu }{(1)}=0.6$$ and $$T_{rP+sQ}^{\mu }{(0)}=1$$, $$T_{rP+sQ}^{\mu }{(1)}=0.6$$.

- Case 1: Let $m=0,~n=0$ and $r, s \in R=Z_{2}$, clearly $T^{\mu }_{S}(r0 + s0)=1 \ge T_{P}^{\mu }{(0)} \wedge T_{Q}^{\mu }(0)=1$.
- Case 2: Let $m=0,~n=1$ and $r, s \in R=Z_{2}$, clearly $T^{\mu }_{S}(r0 + s1)=1 ~or~ 0.6 \ge T_{P}^{\mu }{(0)} \wedge T_{Q}^{\mu }(1)=0.6$.
- Case 3: Let $m=1,~n=0$ and $r, s \in R=Z_{2}$, clearly $T^{\mu }_{S}(r1 + s0)=1 ~or~ 0.6 \ge T_{P}^{\mu }{(1)} \wedge T_{Q}^{\mu }(0)=0.6$.
- Case 4: Let $m=1,~n=1$ and $r, s \in R=Z_{2}$, clearly $T^{\mu }_{S}(r1 + s1)=1 ~or~ 0.6 \ge T_{P}^{\mu }{(1)} \wedge T_{Q}^{\mu }(0)=0.6$.

In all cases we can see that $T^{\mu }_{S}(rm + sn) \ge T_{P}^{\mu }{(m)} \wedge T_{Q}^{\mu }(n),~\forall ~ m,n \in M$

$$\Leftrightarrow$$ $$T_{rP+sQ}^{\mu }{(0)}=1 \le T_{S}^{\mu }{(0)}=1,~and~ T_{rP+sQ}^{\mu }{(1)}=0.6 \le T_{S}^{\mu }{(1)}=0.6$$.

$\Rightarrow$ ${(\mu ,\nu ,\omega )}$-svnss *P*, *Q* and *S* satisfy the condition

(1) $T^{\mu }_{S}(rm + sn) \ge T_{P}^{\mu }{(m)} \wedge T_{Q}^{\mu }(n)$, for all $m, n\in M$ if and only if $T^{\mu }_{rP+sQ} \le T^{\mu }_{S}$.

Similarly we can show for the other clauses i.e indeterminacy membership as well as falsity membership.

### Theorem 3.23

Let *P* be a ${(\mu ,\nu ,\omega )}$-svns on *M* and $r, s \in R$. Then the following conditions must holds;

1. $$T^{\mu }_{rP} \le T_{P}^{\mu }$$ $$\Leftrightarrow$$ $$T_{P}^{\mu }(rm) \ge T_{P}^{\mu }{(m)}$$,
  $$I_{rP}^{\nu } \le I_{P}^{\nu }$$ $$\Leftrightarrow$$ $$I_{P}^{\nu }(rm) \ge I_{P}^{\nu }{(m)}$$ and
  $$F_{rP}^{\omega } \ge F_{P}^{\omega }$$ $$\Leftrightarrow$$ $$F_{P}^{\omega }(rm) \le F_{P}^{\omega }{(m)}$$, for each $$m\in M$$.
2. $$T_{rP+sP}^{\mu } \le T_{P}^{\mu } \Leftrightarrow T_{P}^{\mu }(rm + sn) \ge T_{P}^{\mu }{(m)} \wedge T_{P}^{\mu }(n)$$,
  $$I_{rP+sP}^{\nu } \le I_{P}^{\nu } \Leftrightarrow I_{P}^{\nu }(rm + sn) \ge I_{P}^{\nu }{(m)} \wedge I_{P}^{\nu }(n)$$,
  $$F_{rP+sP}^{\omega } \ge F_{P}^{\omega } \Leftrightarrow F_{P}^{\omega }(rm + sn) \le F_{P}^{\omega }{(m)} \vee F_{P}^{\omega }(n)$$.

### *Proof*

It is easy to prove with the help of Proposition 3.17 and 3.21. $\square$

### Theorem 3.24

Let *P* be a ${(\mu ,\nu ,\omega )}$-svns on *M*. Then *P* is a svnsm of *M* $\Leftrightarrow$ *P* is a single-valued neutrosophic subgroup of the additive group *M*, in the notion of^35^, and meets the requirements $T_{rP}^{\mu } \le T_{P}^{\mu }, ~I_{rP}^{\nu } \le I_{P}^{\nu }$ and $F_{rP}^{\omega } \ge F_{P}^{\omega }$, for every $r\in R$.

### *Proof*

From the description of a single-valued neutrosophic subgroup in^35^ also using Theorem 3.23, it is easy to proof. $\square$

### Theorem 3.25

Assume that *P* is a $(\mu ,\nu ,\omega )$-svns on *M*. Then $P \in svnsm(M)$ $\Leftrightarrow$ the characteristic below are hold:

1. $$P^{(\mu ,\nu ,\omega )}(0) = {{\tilde{X}}}$$.
2. $$P^{(\mu ,\nu ,\omega )}(rm + sn) \ge P^{(\mu ,\nu ,\omega )}{(m)} \wedge P^{(\mu ,\nu ,\omega )}(n)$$, for every $$m, n \in M$$, $$r, s \in R$$.

### *Proof*

Assume that *P* is a ${(\mu ,\nu ,\omega )}$-svnsm of *M* and $e, f \in M$. It is clearly shows that $P^{(\mu ,\nu ,\omega )}(0) = {{\tilde{X}}}$ by using the condition (M1) of Definition 3.8. The foregoing statements are also correct based on (M2) and (M3).

$$\begin{aligned} T_{P}^{\mu }(r m + s n)\ge & {} T_{P}^{\mu }(r m) \wedge T_{P}^{\mu }(s n) \ge T_{P}^{\mu }{(m)} \wedge T_{P}^{\mu }(n),\\ I_{P}^{\nu }(r m + s n)\ge & {} I_{P}^{\nu }(r m) \wedge I_{P}^{\nu }(s n) \ge I_{P}^{\nu }{(m)} \wedge I_{P}^{\nu }(n),\\ F_{P}^{\omega }(r m+ s n)\le & {} T_{P}^{\mu }(r m) \vee F_{P}^{\omega }(s n) \le F_{P}^{\omega }{(m)} \vee F_{P}^{\omega }(n), ~\forall ~ m, n \in M, r, s \in R. \end{aligned}$$

Hence,

$$\begin{aligned} P^{(\mu ,\nu ,\omega )}(r m + s n)&= (T_{P}^{\mu }(r m + s n), I_{P}^{\nu }(r m + s n), F_{P}^{\omega }(r m + s n))\\\ge & {} (T_{P}^{\mu }{(m)} \wedge T_{P}^{\mu }(n), I_{P}^{\nu }{(m)} \wedge I_A{}(n), F_{P}^{\omega }{(m)} \vee F_{P}^{\omega }(n))\\&= (T_{P}^{\mu }{(m)}, I_{P}^{\nu }{(m)}, F_{P}^{\omega }{(m)}) \wedge (T_{P}^{\mu }(n), I_{P}^{\nu }(n), F_{P}^{\omega }(n))\\&= P^{(\mu ,\nu ,\omega )}{(m)}\wedge P^{(\mu ,\nu ,\omega )}(n). \end{aligned}$$

$$\Rightarrow ~~P^{(\mu ,\nu ,\omega )}(rm + sn) \ge P^{(\mu ,\nu ,\omega )}{(m)}\wedge P^{(\mu ,\nu ,\omega )}(n).$$

Conversely, assume $P^{(\mu ,\nu ,\omega )}$ meets the conditions (i) and (ii). Therefore the assumption is evident that $P^{(\mu ,\nu ,\omega )}(0) = {{\tilde{X}}}$.

$$\begin{aligned} T_{P}^{\mu }(m + n)&= T_{P}^{\mu }(1.m + 1.n)\ge T_{P}^{\mu }{(m)} \wedge T_{P}^{\mu }(n),\\ I_{P}^{\nu }(m + n)&= I_{P}^{\nu }(1.m + 1.n) \ge I_{P}^{\nu }{(m)} \wedge I_{P}^{\nu }(n),\\ F_{P}^{\omega }(m + n)&= F_{P}^{\omega }(1.m + 1.m) \le F_{P}^{\omega }{(m)} \vee F_{P}^{\omega }(n). \end{aligned}$$

So, $$P^{(\mu ,\nu ,\omega )}(m + n) \ge P^{(\mu ,\nu ,\omega )}(m) \wedge P^{(\mu ,\nu ,\omega )}(n).$$

Furthermore, the requirement (M2) of Definition 3.8 is fulfilled. Let us now demonstrate the condition’s legitimacy (M3). According to the hypothesis,

$$\begin{aligned} T_{P}^{\mu }(rm)&= T_{P}^{\mu }(rm + r0) \ge T_{P}^{\mu }{(m)} \wedge T_{P}^{\mu }(0) = T_{P}^{\mu }{(m)},\\ I_{P}^{\nu }(rm)&= I_{P}^{\nu }(rm + r0) \ge I_{P}^{\nu }{(m)} \wedge I_{P}^{\nu }(0) = I_{P}^{\nu }{(m)},\\ F_{P}^{\omega }(rm)&= F_{P}^{\omega }(rm + r0) \le F_{P}^{\omega }{(m)} \vee F_{P}^{\omega }(0) = F_{P}^{\omega }{(m)},~~ \forall ~m, n \in M, r \in R. \end{aligned}$$

As a result, (M3) of Definition 3.8 is achieved. $\square$

### Theorem 3.26

Assume *P* and *Q* are ${(\mu ,\nu ,\omega )}$-svnsm of a classical module *M*, then $P ~\cap ~ Q$ is also a ${(\mu ,\nu ,\omega )}$-svnsm of *M*.

### *Proof*

Since $P, Q \in {(\mu ,\nu ,\omega )}$-svnsm(M), we have $P^{(\mu ,\nu ,\omega )}(0) = {{\tilde{X}}}$, and $Q^{(\mu ,\nu ,\omega )}(0) = {{\tilde{X}}}$.

$$\begin{aligned} T_{P~\cap ~ Q}^{\mu }(0)&= T_{P}^{\mu }(0) \wedge T_{Q}^{\mu }(0) = 1,\\ I_{P~\cap ~ Q}^{\nu }(0)&= I_{P}^{\nu }(0) \wedge I_{Q}^{\nu }(0) = 1,\\ F_{P~\cap ~ Q}^{\omega }(0)&= F_{P}^{\omega }(0) \vee F_{Q}^{\omega }(0) = 0. \end{aligned}$$

Hence $(P^{(\mu ,\nu ,\omega )}~\cap ~ Q^{(\mu ,\nu ,\omega )})(0) = {{\tilde{X}}}$ and we find that the condition (M1) of Definition 3.8 is met. Let $m, n \in M, r, s \in R$. According to Theorem 3.25, it is sufficient to demonstrate that

$$\begin{aligned} (P^{(\mu ,\nu ,\omega )} ~\cap ~ Q^{(\mu ,\nu ,\omega )})(rm + sn) \ge (P^{(\mu ,\nu ,\omega )} ~\cap ~ Q^{(\mu ,\nu ,\omega )}){(m)} ~\wedge ~ (P^{(\mu ,\nu ,\omega )} ~\cap ~ Q^{(\mu ,\nu ,\omega )})(n). \end{aligned}$$

That is,

$$\begin{aligned} T_{P~\cap ~ Q}^{\mu }(rm+sn)\ge & {} T_{P~\cap ~ Q}^{\mu }{(m)}~\wedge ~ T_{P~\cap ~ Q}^{\mu }(n),\\ I_{P~\cap ~ Q}^{\nu }(rm+sn)\ge & {} I_{P~\cap ~ Q}^{\nu }{(m)}~\wedge ~ I_{P~\cap ~ Q}^{\nu }(n),\\ F_{P~\cap ~ Q}^{\omega }(rm+sn)\le & {} F_{P~\cap ~ Q}^{\omega }{(m)} ~\vee ~ F_{P~\cap ~ Q}^{\omega }(n).\\ \end{aligned}$$

Now consider the truth, indeterminacy and falsity membership degree of the intersection,

$$\begin{aligned} T_{P~\cap ~ Q}^{\mu }(rm+sn)&= T_{P}^{\mu }(rm+sn) \wedge T_{Q}^{\mu }(rm+sn)\\\ge & {} (T_{P}^{\mu }{(m)} \wedge T_{P}^{\mu }(n)) \wedge (T_{Q}^{\mu }{(m)} \wedge T_{Q}^{\mu }(n))\\&= (T_{P}^{\mu }{(m)} \wedge T_{Q}^{\mu }{(m)}) \wedge (T_{P}^{\mu }(n) \wedge T_{Q}^{\mu }(n))\\&= T_{P~\cap ~ Q}^{\mu }{(m)} \wedge T_{P~\cap ~ Q}^{\mu }(n).\\ \Rightarrow ~~ T_{P~\cap ~ Q}^{\mu }(rm+sn)\ge & {} T_{P~\cap ~ Q}^{\mu }{(m)} \wedge T_{P~\cap ~ Q}^{\mu }(n)\\ I_{P~\cap ~ Q}^{\nu }(r m + s n)&= I_{P}^{\mu }(rm + sn) \wedge T_{Q}^{\nu }(rm + sn)\\\ge & {} (I_{P}^{\nu }{(m)} \wedge I_{P}^{\nu }(n)) \wedge (T_{Q}^{\nu }{(m)} \wedge I_{Q}^{\nu }(n))\\&= (I_{P}^{\mu }{(m)} \wedge T_{Q}^{\nu }{(m)}) \wedge (I_{P}^{\mu }(n) \wedge I_{Q}^{\mu }(n))\\&= I_{P~\cap ~ Q}^{\nu }{(m)} \wedge T_{P~\cap ~ Q}^{\mu }(n).\\ \Rightarrow ~~ I_{P~\cap ~ Q}^{\nu }(rm+sn)\ge & {} I_{P~\cap ~ Q}^{\nu }{(m)} \wedge I_{P~\cap ~ Q}^{\nu }(n)\\ F_{P~\cap ~ Q}^{\omega }(r m + s n)&= F_{P}^{\omega }(rm + sn) \vee I_{Q}^{\omega }(rm + sn)\\\le & {} (F_{P}^{\omega }{(m)} \vee F_{P}^{\omega }(n)) \vee (F_{Q}^{\omega }{(m)} \vee F_{Q}^{\omega }(n))\\&= (F_{P}^{\omega }{(m)} \vee F_{Q}^{\omega }{(m)}) \vee (F_{P}^{\omega }(n) \vee F_{Q}^{\omega }(n))\\&= F_{P~\cap ~ Q}^{\omega }{(m)} \vee F_{P~\cap ~ Q}^{\omega }(n).\\ \Rightarrow F_{P~\cap ~ Q}^{\omega }(rm+sn)\le & {} F_{P~\cap ~ Q}^{\omega }{(m)} \vee F_{P~\cap ~ Q}^{\omega }(n). \end{aligned}$$

Hence, $$P ~\cap ~ Q \in (\mu ,\nu ,\omega )$$-*svnsm*(*M*). $$\square$$

Note: Let *N* be a nonempty subset of *M* is a submodule of *M* $\Leftrightarrow$ $rm + sn \in N,~ \forall ~m,~n \in M,~r,~s \in R$.

### Proposition 3.27

Suppose *M* is a module over *R*. $P \in {(\mu ,\nu ,\omega )}$-svnsm(M) $\Leftrightarrow$ $\forall$ $\alpha \in [0, 1]$, $\alpha$-level sets of $P^{(\mu ,\nu ,\omega )}$,  $(T_{P}^{\mu })_{\alpha }, ~(I_{P}^{\nu })_{\alpha }$ and $(F_{P}^{\omega })^{\alpha }$ are classical submodules of *M* where $P^{(\mu ,\nu ,\omega )}(0) = {{\tilde{X}}}$.

### *Proof*

Let $P \in {(\mu ,\nu ,\omega )}$-svnsm(M), $\alpha \in [0, 1]$, $m, n \in (T_{P}^{\mu })_{\alpha }$ and $r, s \in R$ can represent a certain element. Then

$$\begin{aligned} T_{P}^{\mu }{(m)} \ge \alpha , ~T_{P}^{\mu }(n) \ge \alpha ~\text {and}~ T_{P}^{\mu }{(m)} \wedge T_{P}^{\mu }(n) \ge \alpha . \end{aligned}$$

By using Theorem 3.25, we have

$$\begin{aligned} T_{P}^{\mu }(rm + sn) \ge T_{P}^{\mu }{(m)} \wedge T_{P}^{\mu }(n) \ge \alpha . \end{aligned}$$

Hence,

$$\begin{aligned} rm + sn \in (T_{P}^{\mu })_{\alpha }. \end{aligned}$$

As a result, with each $\alpha \in [0, 1]$, $(T_{P}^{\mu })_{\alpha }$ is a classical submodule of *M*. Similarly, for $m, n \in (I_{P}^{\nu })_{\alpha }, ~(F_{P}^{\omega })^{\alpha }$ we obtain $rm+sn \in (I_{P}^{\nu })_{\alpha }, ~(F_{P}^{\omega })^{\alpha }$ for each $\alpha \in [0, 1]$. Consequently, $(I_{P}^{\nu })_{\alpha }, ~(F_{P}^{\omega })^{\alpha }$ with each $\alpha \in [0, 1]$ are classical submodules of *M*.

Conversely, let $(T_{P}^{\mu })_{\alpha }$ with each $\alpha \in [0, 1]$ be a classical submodules of *M*.

Let $m, n \in M$, $\alpha = T_{P}^{\mu }{(m)} \wedge T_{P}^{\mu }(n)$. Then $T_{P}^{\mu }{(m)} = \alpha$ and $T_{P}^{\mu }(n) = \alpha$. Thus, $m, n \in (T_{P}^{\mu })_{\alpha }$.

Since $(T_{P}^{\mu })_{\alpha }$ is a classical submodule of *M*, so we have $r m + s n \in (T_{P}^{\mu })_{\alpha }$ for all $r, s \in R$.

$$\begin{aligned} \Rightarrow ~(T_{P}^{\mu })(rm + sn) \ge \alpha = T_{P}^{\mu }{(m)} \wedge T_{P}^{\mu }(n). \end{aligned}$$

Similarly, $(I_{P}^{\nu })_{\alpha }$ with each $\alpha \in [0, 1]$ be a classical submodules of *M*.

Let $m, n \in M$, $\alpha = I_{P}^{\nu }{(m)} \wedge I_{P}^{\nu }(n)$. Then $I_{P}^{\nu }{(m)} = \alpha$ and $I_{P}^{\nu }(n) = \alpha$. Thus, $m, n \in (I_{P}^{\nu })_{\alpha }$.

Since $(I_{P}^{\nu })_{\alpha }$ is a classical submodule of *M*, so we have $r m + s n \in (I_{P}^{\nu })_{\alpha }$ for all $r, s \in R$.

$$\begin{aligned} \Rightarrow ~(I_{P}^{\nu })(rm + sn) \ge \alpha = I_{P}^{\nu }{(m)} \wedge I_{P}^{\nu }(n). \end{aligned}$$

Now we consider $(F_{P}^{\omega })^{\alpha }$. Let $m, n \in M$, $\alpha = F_{P}^{\omega }{(m)}\vee F_{P}^{\omega }(n)$. Then $F_{P}^{\omega }{(m)} = \alpha$, $F_{P}^{\omega }(n) = \alpha$.

Thus $m, n \in (F_{P}^{\omega })^{\alpha }$. Since $(F_{P}^{\omega })^{\alpha }$ is a submodule of *M*, we have $rm + sn \in (F_{P}^{\omega })^{\alpha }$ for all $r, s \in R$.

Thus $(F_{P}^{\omega })(rm + sn) \le \alpha = F_{P}^{\omega }{(m)} \vee F_{P}^{\omega }(n)$. It is also obvious that $P^{(\mu ,\nu ,\omega )}(0) = {\tilde{X}}$.

As a result, the conditions of Theorem 3.25 are fulfilled. $\square$

### Proposition 3.28

Assume that *P* and *Q* are two $(\mu ,\nu ,\omega )$-svnss on *X* and *Y*, respectively. Then, for the $\alpha$- levels, the following equalities are hold.

$$\begin{aligned} (T_{P\times Q}^{\mu })_{\alpha }&= (T_{P}^{\mu })_{\alpha } \times (T_{Q}^{\mu })_{\alpha },\\ (I_{P\times Q}^{\nu })_{\alpha }&= (I_{P}^{\nu })_{\alpha } \times (I_{Q}^{\nu })_{\alpha },\\ (F_{P\times Q}^{\omega })^{\alpha }&= (F_{P}^{\omega })^{\alpha } \times (F_{Q}^{\omega })^{\alpha }. \end{aligned}$$

### *Proof*

Let $(m, n) \in (T_{P\times Q}^{\mu })_{\alpha }$ be arbitrary.

So,

$$\begin{aligned}&T_{P\times Q}^{\mu }(m, n) \ge \alpha \Leftrightarrow T_{P}^{\mu }{(m)} \wedge T_{Q}^{\mu }(n) \ge \alpha ,\\&\Leftrightarrow T_{P}^{\mu }{(m)}\ge \alpha ,~T_{P}^{\mu }(n)\ge \alpha \Leftrightarrow (m, n) \in (T_{P}^{\mu })_\alpha \times (T_{Q}^{\mu })_\alpha . \end{aligned}$$

Now Let $(m, n) \in (I_{P\times Q}^{\nu })_{\alpha }$ be arbitrary.

So,

$$\begin{aligned}&I_{P\times Q}^{\nu }(m, n)\ge \alpha \Leftrightarrow I_{P}^{\nu }{(m)} \wedge I_{Q}^{\nu }(n) \ge \alpha ,\\&\Leftrightarrow I_{P}^{\nu }{(m)}\ge \alpha ,~I_{P}^{\nu }(n)\ge \alpha , \Leftrightarrow (m, n) \in (I_{P}^{\nu })_\alpha \times (T_{Q}^{\nu })_\alpha . \end{aligned}$$

Similarly $(m, n) \in (F_{P\times Q}^{\omega })^{\alpha }$ be arbitrary.

So,

$$\begin{aligned}&F_{P\times Q}^{\omega }(m, n) \le \alpha \Leftrightarrow F_{P}^{\omega }{(m)} \vee F_{Q}^{\omega }(n) \le \alpha ,\\&\Leftrightarrow F_{P}^{\omega }{(m)}\le \alpha ,~F_{P}^{\omega }(n)\le \alpha \Leftrightarrow (m, n) \in (F_{P}^{\omega })^\alpha \times (F_{Q}^{\omega })^\alpha . \end{aligned}$$

$$\square$$

### Proposition 3.29

Let *P* and *Q* be two ${(\mu ,\nu ,\omega )}$-svnss on *X* and *Y*, respectively and $g : X \rightarrow Y$ be a mapping. Therefore the preceding must be applicable:

1. $$\begin{aligned} g((T_{P}^{\mu }))_{\alpha } \subseteq (T_{g(P)}^{\mu })_{\alpha }, \\ g((I_{P}^{\nu })_{\alpha }) \subseteq (I_{g(P)}^{\nu })_{\alpha }, \\ g((F_{P}^{\omega })^{\alpha })\supseteq (F_{g(P)}^{\omega })^{\alpha }. \end{aligned}$$
2. $$\begin{aligned} g^{-1}((T_{Q}^{\mu })_{\alpha })= (T_{g^{-1}(Q)}^{\mu })_{\alpha }, \\ g^{-1}((I_{Q}^{\nu })_{\alpha }) = (I_{g^{-1}(Q)}^{\nu })_{\alpha }, \\ g^{-1}((F_{Q}^{\omega })^{\alpha })= (F_{g^{-1}(Q)}^{\omega })^{\alpha }. \end{aligned}$$

### *Proof*

(1) Let $n \in g((T_{P}^{\mu })_{\alpha })$. Then $\exists$ $m\in (T_{P}^{\mu })_{\alpha }$ such that $g{(m)} = n$. Hence $T_{P}^{\mu }{(m)} \ge \alpha$.

So, $\bigvee \limits _{m\in g^{-1}(n)}^{} T_{P}^{\mu }{(m)} \ge \alpha$. That is, $T_{g(P)}^{\mu }(n) \ge \alpha$ and $n \in (T_{g(P)}^{\mu })_{\alpha }$. Hence $g((T_{P}^{\mu })_{\alpha }) \subseteq (T_{g(P)}^{\mu })_{\alpha }$.

Similarly, $n \in g((I_{P}^{\nu })_{\alpha })$. Then $\exists$ $m\in (I_{P}^{\nu })_{\alpha }$ such that $g{(m)} = n$. Thus $I_{P}^{\nu }{(m)} \ge \alpha$.

So, $\bigvee \limits _{m\in g^{-1}(n)}^{} I_{P}^{\nu }{(m)} \ge \alpha$. That is, $I_{g(P)}^{\nu }(n) \ge \alpha$ and $n \in (I_{g(P)}^{\nu })_{\alpha }$. So $g((I_{P}^{\nu })_{\alpha }) \subseteq (I_{g(P)}^{\nu })_{\alpha }$.

Also, $n \in g((F_{P}^{\omega })^{\alpha })$. Then $\exists$ $m\in (F_{P}^{\omega })^{\alpha }$ such that $g{(m)} = n$. This implies $F_{P}^{\omega }{(m)} \le \alpha$.

So, $\bigwedge \limits _{m\in g^{-1}(n)}^{} F_{P}^{\omega }{(m)} \le \alpha$. That is, $F_{g(P)}^{\omega }(n) \le \alpha$ and $n \in (F_{g(P)}^{\omega })^{\alpha }$. Hence $g((F_{P}^{\omega })^{\alpha }) \supseteq (F_{g(P)}^{\omega })_{\alpha }$.

(2)

$$\begin{aligned} (T_{g^{-1}(Q)}^{\mu })_{\alpha }&= \{m\in X : T_{g^{-1}(Q)}^{\mu }{(m)} \ge \alpha \}\\&= \{m\in X : T_{Q}^{\mu }(g{(m)}) \ge \alpha \}\\&= \{m\in X : g{(m)} \in (T_{Q}^{\mu })_{\alpha }\}\\&= \{m\in X : m\in g^{-1}((T_{Q}^{\mu })_\alpha )\}\\&= g^{-1}((T_{Q}^{\mu })_{\alpha }). \end{aligned}$$

Similarly,

$$\begin{aligned} (I_{g^{-1}(Q)}^{\nu })_{\alpha }&= \{m\in X : I_{g^{-1}(Q)}^{\nu }{(m)} \ge \alpha \}\\&= \{m\in X : I_{Q}^{\nu }(g{(m)}) \ge \alpha \}\\&= \{m\in X : g{(m)} \in (I_{Q}^{\nu })_{\alpha }\}\\&= \{m\in X : m\in g^{-1}((I_{Q}^{\nu })_\alpha )\}\\&= g^{-1}((I_{Q}^{\nu })_{\alpha }). \end{aligned}$$

Also,

$$\begin{aligned} (F_{g^{-1}(Q)}^{\omega })^{\alpha }&= \{m\in X : F_{g^{-1}(Q)}^{\omega }{(m)} \le \alpha \}\\&= \{m\in X : F_{Q}^{\omega }(g{(m)}) \le \alpha \}\\&= \{m\in X : g{(m)} \in (F_{Q}^{\omega })^{\alpha }\}\\&= \{m\in X : m\in g^{-1}((F_{Q}^{\omega })^\alpha )\}\\&= g^{-1}((F_{Q}^{\omega })^{\alpha }). \end{aligned}$$

$$\square$$

### Theorem 3.30

Let $P, ~Q \in {(\mu ,\nu ,\omega )}$-svnsm(M). Then the product $P \times Q$ is also a ${(\mu ,\nu ,\omega )}$-svnsm(M) of *M*.

### *Proof*

We know that direct product of two submodule is a module. By using Proposition 3.27, suppose *M* is a module over *R*. $P \in {(\mu ,\nu ,\omega )}$-svnsm(M) $\Leftrightarrow$ $\forall$ $\alpha \in [0, 1]$, $\alpha$-level sets of $P^{(\mu ,\nu ,\omega )}$,  $(T_{P}^{\mu })_{\alpha }, ~(I_{P}^{\nu })_{\alpha }$ and $(F_{P}^{\omega })^{\alpha }$ are classical submodules of *M* where $P^{(\mu ,\nu ,\omega )}(0) = {{\tilde{X}}}$. Also by using Proposition 3.28, assume that *P* and *Q* are two $(\mu ,\nu ,\omega )$-svnss on *X* and *Y*, respectively. Then, for the $\alpha$- levels, the following equalities are hold.

$$\begin{aligned} (T_{P\times Q}^{\mu })_{\alpha }&= (T_{P}^{\mu })_{\alpha } \times (T_{Q}^{\mu })_{\alpha },\\ (I_{P\times Q}^{\nu })_{\alpha }&= (I_{P}^{\nu })_{\alpha } \times (I_{Q}^{\nu })_{\alpha },\\ (F_{P\times Q}^{\omega })^{\alpha }&= (F_{P}^{\omega })^{\alpha } \times (F_{Q}^{\omega })^{\alpha }. \end{aligned}$$

So, by combining these two result we get that $P \times Q$ is a ${(\mu ,\nu ,\omega )}$-svnsm(M) of *M*. $\square$

### Theorem 3.31

Assume $g : M \rightarrow N$ be a homomorphism of modules whereas *M*,  *N* be the classical modules. If *P* is a ${(\mu ,\nu ,\omega )}$-svnsm of *M*, then the image *g*(*P*) is a ${(\mu ,\nu ,\omega )}$-svnsm of *N*.

### *Proof*

It is sufficient to prove by Proposition 3.27,

$$\begin{aligned} (T_{g(P)}^{\mu })_{\alpha }, ~(I_{g(P)}^{\nu })_{\alpha }, (F_{g(P)}^{\omega })^{\alpha } \end{aligned}$$

are ${(\mu ,\nu ,\omega )}$-svnsm of *N*, $\forall$ ${\alpha } \in [0, 1]$.

Let $n_{1}, n_{2} \in (T_{g(P)}^{\mu })_{\alpha }$. Then $T_{g(P)}^{\mu }(n_{1}) \ge \alpha$ and $T_{g(P)}^{\mu }(n_{2}) \ge \alpha$. There exist $m_{1},~m_{2} \in M$ such that

$$\begin{aligned} T_{P}^{\mu }(m_{1}) \ge T_{g(P)}^{\mu }(n_{1}) \ge \alpha ~\text {and}~ T_{P}^{\mu }(m_{2}) \ge T_{g(P)}^{\mu }(n_{2}) \ge \alpha . \end{aligned}$$

So,

$$\begin{aligned} T_{P}^{\mu }(m_{1}) \ge \alpha ,~ T_{P}^{\mu }(m_{2}) \ge \alpha ~\text {and}~ T_{P}^{\mu }(m_{1}) \wedge T_{P}^{\mu }(m_{2}) \ge \alpha . \end{aligned}$$

Since *P* is a ${(\mu ,\nu ,\omega )}$-svnsm of *M*, for any $r, s \in R$, we have

$$\begin{aligned} T_{P}^{\mu }(rm_{1} + sm_{2}) \ge T_{P}^{\mu }(m_{1}) \wedge T_{P}^{\mu }(m_{2}) \ge \alpha . \end{aligned}$$

Hence,

$$\begin{aligned} & rm_{1} + sm_{2} \in (T_{P}^{\mu })_{\alpha }. \\&\Rightarrow ~~g(rm_{1} + sm_{2}) \in g((T_{P}^{\mu })_{\alpha }) \subseteq (T_{g(P)})_{\alpha }\\&\Rightarrow ~~ rg(m_{1}) + sg(m_{2}) \in (T_{g(P)})_{\alpha }\\&\Rightarrow ~~rn_{1} + sn_{2} \in (T_{g(P)}^{\mu })_{\alpha }. \end{aligned}$$

Therefore, $(T_{g(P)}^{\mu })_{\alpha }$ is a submodule of *N*.

Similarly, $\forall$ $\alpha \in [0, 1]$, consider $n_{1}, n_{2} \in (I_{g(P)}^{\nu })_{\alpha }$. Then $I_{g(P)}^{\nu }(n_{1}) \ge \alpha$ and $I_{g(P)}^{\nu }(n_{2}) \ge \alpha$. There exist $m_{1},~m_{2} \in M$ such that

$$\begin{aligned} I_{P}^{\nu }(m_{1}) \ge I_{g(P)}^{\nu }(n_{1}) \ge \alpha ~\text {and}~ I_{P}^{\nu }(m_{2}) \ge I_{g(P)}^{\nu }(n_{2}) \ge \alpha . \end{aligned}$$

So

$$\begin{aligned} I_{P}^{\nu }(m_{1}) \ge \alpha ,~ I_{P}^{\nu }(m_{2}) \ge \alpha ~\text {and} ~ I_{P}^{\nu }(m_{1}) \wedge I_{P}^{\nu }(m_{2}) \ge \alpha . \end{aligned}$$

Since *P* is a ${(\mu ,\nu ,\omega )}$-svnsm of *M*, for any $r, s \in R$, we have

$$\begin{aligned} I_{P}^{\nu }(rm_{1} + sm_{2}) \ge I_{P}^{\nu }(m_{1}) \wedge I_{P}^{\nu }(m_{2}) \ge \alpha . \end{aligned}$$

Hence,

$$\begin{aligned} & rm_{1} + sm_{2} \in (I_{P}^{\nu })_{\alpha }). \\&\Rightarrow ~~~g(rm_{1} + sm_{2}) \in g((I_{P}^{\nu })_{\alpha }) \subseteq (I_{g(P)})_{\alpha }\\&\Rightarrow ~~~rg(m_{1}) + sg(m_{2}) \in (I_{g(P)})_{\alpha }\\&\Rightarrow ~~~rn_{1} + sn_{2} \in (I_{g(P)}^{\nu })_{\alpha }. \end{aligned}$$

Therefore, $(I_{g(P)}^{\nu })_{\alpha }$ is a submodule of *N*.

Similarly, for all $\alpha \in [0, 1]$, consider $n_{1}, n_{2} \in (n_{g(P)}^{\omega })^{\alpha }$. Then $n_{g(P)}^{\omega }(n_{1}) \le \alpha$ and $n_{g(P)}^{\omega }(n_{2}) \le \alpha$. There exist $m_{1},~m_{2} \in M$ such that

$$\begin{aligned} F_{P}^{\omega }(m_{1}) \le F_{g(P)}^{\omega }(n_{1}) \le \alpha \end{aligned}$$

and

$$\begin{aligned} F_{P}^{\omega }(m_{2}) \le F_{g(P)}^{\omega }(n_{2}) \le \alpha . \end{aligned}$$

So $F_{P}^{\omega }(m_{1}) \le \alpha$, $F_{P}^{\omega }(m_{2}) \le \alpha$ and $F_{P}^{\omega }(m_{1}) \vee F_{P}^{\omega }(m_{2}) \le \alpha$. Since *P* is a ${(\mu ,\nu ,\omega )}$-svnsm of *M*, for any $r, s \in R$, we have $F_{P}^{\omega }(rm_{1} + sm_{2}) \le F_{P}^{\omega }(m_{1}) \vee F_{P}^{\omega }(m_{2}) \le \alpha$.

Hence,

$$\begin{aligned} & rm_{1} + sm_{2} \in (F_{P}^{\omega })_{\alpha }). \\&\Rightarrow ~~~g(rm_{1} + sm_{2}) \in g((F_{P}^{\omega })^{\alpha }) \supseteq (F_{g(P)})^{\alpha }\\&\Rightarrow ~~~ rg(m_{1}) + sg(m_{2}) \in (F_{g(P)})^{\alpha } \\&\Rightarrow ~~~~~rn_{1} + sn_{2} \in (F_{g(P)}^{\omega })^{\alpha }. \end{aligned}$$

Therefore, $(F_{g(P)}^{\omega })^{\alpha }$ is a submodule of *N*. So, for every $\alpha \in [0, 1]$, $(T_{g(P)}^{\mu })_{\alpha }$ $(I_{g(P)}^{\nu })_{\alpha }$, $(F_{g(P)}^{\omega })^{\alpha }$ are classical submodules of *N*. Thus *g*(*P*) is a ${(\mu ,\nu ,\omega )}$-svnsm of *N* via the use of Proposition 3.27. $\square$

### Theorem 3.32

Assume $g : M \rightarrow N$ be a homomorphism of modules whereas *M*,  *N* be the classical modules. If *Q* is a ${(\mu ,\nu ,\omega )}$-svnsm of *N*, then the preimage $g^{-1}(Q)$ is a ${(\mu ,\nu ,\omega )}$-svnsm of *M*.

### *Proof*

Using Proposition 3.29 (2), we have

$$\begin{aligned} g^{-1}((T_{Q}^{\mu })_{\alpha })&= (T_{g^{-1}(Q)}^{\mu })_{\alpha },\\ ~g^{-1}((I_{Q}^{\nu })_{\alpha })&= (I_{g^{-1}(Q)}^{\nu })_{\alpha },\\ g^{-1}((F_{Q}^{\omega })^{\alpha })&= (F_{g^{-1}(Q)}^{\omega })^{\alpha }. \end{aligned}$$

Since preimage of a ${(\mu ,\nu ,\omega )}$-svnsm is a ${(\mu ,\nu ,\omega )}$-svnsm, by Proposition 3.27 we arrive at a conclusion. $\square$

### Corollary 3.33

If $g : M \rightarrow N$ is a surjective module homomorphism and $\{P_{i} : i \in I\}$ is a family of ${(\mu ,\nu ,\omega )}$-svnsm of *M*, then $g(\cap P_{i})$ is a ${(\mu ,\nu ,\omega )}$-svnsm of *N*.

### Corollary 3.34

If $g : M \rightarrow N$ is a homomorphism of modules and $\{Q_{j} : j \in I\}$ is a family of ${(\mu ,\nu ,\omega )}$-svnsm of *N*, then $g^{-1}(~\cap ~ Q_{j})$ is a ${(\mu ,\nu ,\omega )}$-svnsm of *M*.

## Conclusions

A $(\mu ,\nu ,\omega )$-svns is a type of svns that can be used to tackle real-world challenges in research, engineering, denoising, clustering, segmentation, and a range of medical image-processing applications. Therefore, the study of $(\mu ,\nu ,\omega )$-svnss and their characteristics has a significant influence, both in terms of gaining an understanding of the fundamentals of vulnerability and the applications that can benefit from this knowledge. As a continuation of the research that was carried out in^35–38^, we intend to propose and investigate the idea of a $(\mu ,\nu ,\omega )$-svnsm. In this article, we defined $(\mu ,\nu ,\omega )$-svnm and $(\mu ,\nu ,\omega )$-svnsm and offered a number of fundamental results that are connected to these ideas. As a consequence of this, the purpose of this study is to make use of a variety of different concepts in order to acquire some pertinent outcomes about $(\mu ,\nu ,\omega )$-svnsm that are of significant worth in the field of research. In the realm of algebraic structure theory, it possesses a fantastic novel idea that has the potential to be utilized in the future for the solution of a variety of algebraic issues.

- This approach is frequently extended to the generators of arbitrary nonempty families of neutrosophic submodules as well as structure maintaining features such as isomorphism of neutrosophic submodules. Neutrosophic submodules give a solid mathematical framework for clarifying related scientific issues in image processing, control theory, and economics.
- This notion can be expanded to soft neutrosophic modules, weak soft neutrosophic modules, strong soft neutrosophic modules, soft neutrosophic module homomorphism, and soft neutrosophic module isomorphism. Furthermore, scholars might explore the homological properties of these modules.
- This study can be broadened to include the cyclic fuzzy neutrosophic normal soft group, neutrosophic rings, and ideals.
- In the future, researchers may extend this concept to topological spaces, fields, and vector spaces.

## Data Availability

The data used in the article are hypothetical, and can be used anyone by just citing this article and requests for materials should be addressed to Muhammad Shazib Hameed (shazib.hameed@kfueit.edu.pk).
